# Accuracy of injection and short-term pain relief following intra-articular corticosteroid injection in knee osteoarthritis – an observational study

**DOI:** 10.1186/s12891-017-1401-z

**Published:** 2017-01-26

**Authors:** G. Hirsch, T. W. O’Neill, G. Kitas, A. Sinha, R. Klocke

**Affiliations:** 1 Department of Rheumatology, Dudley Group for Health (DGH) NHS Foundation Trust, Russells Hall Hospital, Dudley, West Midlands DY1 2HQ UK; 2 Faculty of Medical and Human Sciences, Arthritis Research UK Centre for Epidemiology, Institute of Inflammation and Repair, Manchester Academic Health Science Centre, University of Manchester, Manchester, UK; 3 NIHR Manchester Musculoskeletal Biomedical Research Unit, Central Manchester NHS Foundation Trust, Manchester Academic Health Science Centre, Manchester, UK; 4 Department of Radiology, DGH NHS Foundation Trust, Russells Hall Hospital, Dudley, West Midlands UK

**Keywords:** Intra-articular steroid injection, Knee osteoarthritis, Ultrasound, Predictors of response

## Abstract

**Background:**

Intra-articular corticosteroid injections (IACI) are effective treatments for pain in knee osteoarthritis (KOA) but treatment response varies. There is uncertainty as to whether structural factors such as accurate placement of IACI affect outcome. We examined this question in a pragmatic observational study, using ultrasound (US) to verify accuracy of IACI.

**Methods:**

105 subjects with KOA (mean age 63.1 years, 59% female) routinely referred for IACI underwent assessment of demographic factors, x-ray and US of the knee before aspiration and IACI (based on clinical landmarks) with 40 mg triamcinolone acetonide with lignocaine plus a small amount of atmospheric air by an independent physician. US demonstration of intra-articular mobile air, i.e. a positive air arthrosonogram, was used to determine accurate placement of injection. Both patients and injecting physicians were blind to the US findings. Pain at baseline, three and nine weeks post injection was assessed using the 500 mm WOMAC pain subscale and response defined as ≥ 40% reduction in pain from baseline. Inter-observer reliability of air-arthrosonogram assessment was good: κ 0.79 (three raters).

**Results:**

Sixty-three subjects (60.6%) were responders at three weeks and 43 (45.7%) at nine weeks. Seventy-four subjects (70.5%) had a positive arthrosonogram. A positive air arthrosonogram did not associate with a higher rate of response to treatment (p 0.389 at three weeks, p 0.365 at nine weeks). There was no difference in US effusion depth, power Doppler signal or radiographic grade between responders and non-responders to the injection, but female gender associated with response at 3 weeks and previous injection with non-response at 9 weeks.

**Conclusions:**

Accurate intra-articular injection of corticosteroid results did not result in superior outcome in terms of pain compared to inaccurate injection in symptomatic knee OA.

## Background

Osteoarthritis (OA) is the most common form of arthritis worldwide and its prevalence is increasing [1]. Knee osteoarthritis is one of the most common and disabling forms of the condition and has significant clinical and public health impact [2]. In conjunction with other conservative measures to treat pain in knee OA, intra-articular corticosteroid injections (IACI) are commonly used in accordance with published guidance [3, 4]. Meta-analyses of placebo-controlled studies have confirmed that IACI provide effective pain relief for at least three weeks [5, 6], with some studies suggesting effects of 14 weeks or longer [6]. There is considerable variation between individuals in both the magnitude and duration of response and identifying predictors of response to treatment has been suggested a priority for research in the field [5]. Systematic reviews [7, 8] of previous studies addressing the subject have found insufficient evidence of associations between radiographic grade of arthritis [9, 10] or clinical evidence of inflammation [10, 11] and response to IACI. One potentially relevant, but so far insufficiently investigated factor that may plausibly govern response to IACI is accuracy of the injection. While the knee joint is perceived as being easy to inject with a high degree of accuracy, up to a third of routine injections based on anatomical landmarks may fail to enter the joint cavity [12, 13]; it remains unclear whether localisation of the steroid injection to within the knee joint cavity influences outcome.

The aim of this study was to determine whether accuracy of intra-articular placement of the injection, assessed by ultrasound, associates with improved outcome in terms of pain relief following routine IACI, based on clinical landmarks, in knee osteoarthritis.

## Methods

### Subjects

This was a prospective observational cohort study of a series of subjects with symptomatic knee OA who had been recommended by their treating physician to have an intra-articular steroid injection as part of their routine care. The setting was a single teaching district general hospital in the West Midlands of the UK. Potential participants were identified from orthopaedic and rheumatology clinics based in the hospital.

Men and women were eligible if they satisfied the following criteria: i) aged 40 years and over, ii) evidence of osteoarthritis of the knee according to ACR criteria [14], iii) baseline pain of 100/500 mm or higher on the Western Ontario and McMaster Universities Osteoarthritis Index, version 3 (WOMAC) pain subscale [15], and iv) symptoms judged by the referring clinician as meriting IACI. Subjects were excluded if they had received an intra-articular or intramuscular steroid injection within the preceding twelve weeks or were taking oral prednisolone at a dose of 7.5 mg or higher or, a had a diagnosis of fibromyalgia or complex widespread pain, active RA or other inflammatory arthritis

### Baseline assessment

Subjects who consented to the study underwent baseline assessment, immediately prior to treatment. They completed an interviewer-assisted questionnaire which included information about their age, past medical history and whether or not they had received previous knee injection. They also completed the WOMAC questionnaire [15]. This uses five 100 mm visual analogue scales (VAS) to assess knee pain and produces scores with a potential range of 0-500, with higher scores denoting higher levels of pain. Height and weight were assessed in a standard fashion. The baseline assessment also included psychological scales, the details of which will be reported in a separate manuscript. Subjects had radiographs (antero-posterior, lateral and patellofemoral ‘skyline’ views) of their index knee unless already performed within the previous 6 months. High-sensitivity CRP (hsCRP) was measured using an enzyme immunoassay (MP Biomedicals, Solon, Ohio, US).

Radiographs were graded by agreement by two observers (GH and RK), blinded to clinical details, using a standard atlas [16] which allows grading from 0 to 3 [0, normal; 3, severe changes] for individual features of osteophyte and joint space narrowing at both the tibiofemoral and patellofemoral joint (PFJ) including skyline views of the patellofemoral joint. In this grading system, which has been validated previously [17], grade 1 osteophyte is considered equivalent to Kellgren-Lawrence grade 2 [16]. The highest grade for each feature at any site was used for further analysis. In addition, subjects underwent ultrasound assessment with a 5–13 MHz linear US probe (VF13-5; Antares, Siemens Healthcare Diagnostics, Camberley, Surrey, UK) prior to injection for synovial effusion thickness and semi-quantative synovial power Doppler signal estimation as described previously [18, 19]. All ultrasound procedures in the study were performed by GH, a rheumatologist trained and with two years’ experience in MSK US [18].

### Intervention

The intervention was performed by one of several clinicians who were not involved with any of the other study procedures, including ultrasound. Using their usual preferred anatomical approach and injection technique and under guidance of clinical examination and landmarks only, the clinician positioned the patient and they aspirated and injected the joint under aseptic precautions with a 21 G needle and a standard mixture of 40 mg triamcinolone acetonide, 4 ml 1% lignocaine and 2 ml atmospheric air.

### Assessment of accuracy of intra-articular placement of injection

**Fig. 1:**
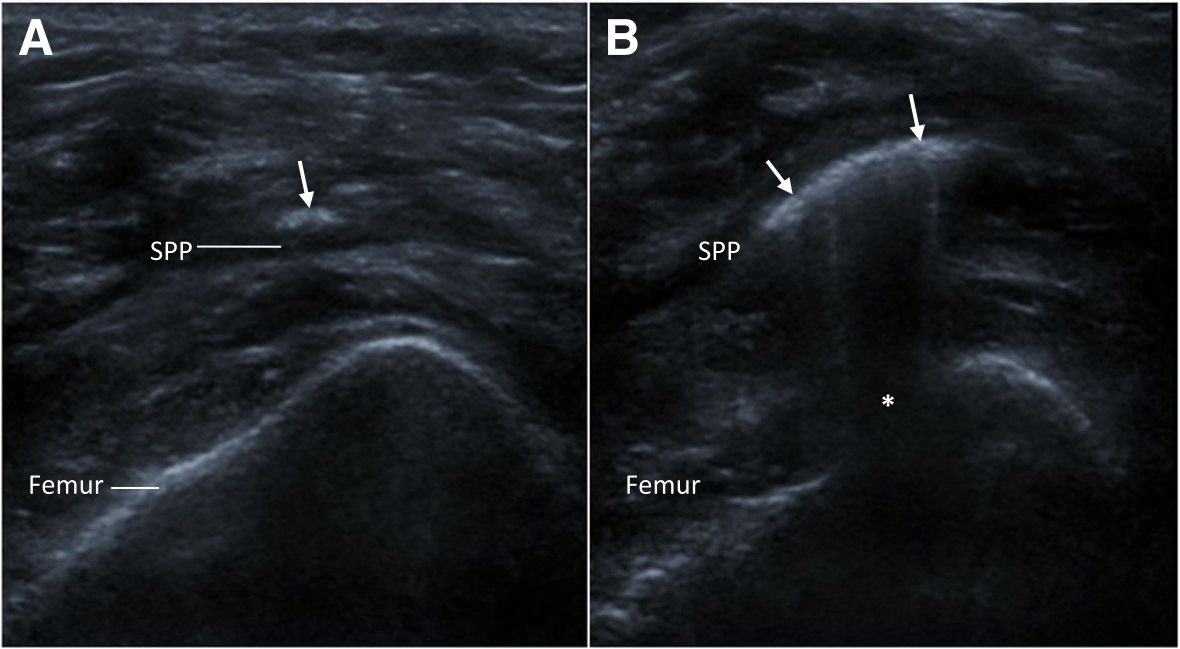
A transverse ultrasound section of the mid supra-patellar pouch area of the right knee during the very early (**a**) and later (**b**) phase of injection, showing a positive air arthrosonogram. There is an emerging area of intra-articular air bubbles (arrows), initially faintly (**a**) and then more clearly visible (**b**), with posterior acoustic shadowing (*). Varying transducer pressure demonstrated the echogenic layer to be displaceable and display buoyancy, consistent with intra-articular placement of the injection. SPP denotes suprapatellar pouch

While the independent clinician performed the injection, the sonographer placed the US probe over the knee joint during the injection to determine placement of the injection. Blinding was maintained: the study subject and injecting clinician were not informed about the US findings. Air provides an effective US contrast medium through its features of being strongly hyperechoic, exhibiting posterior acoustic shadowing, buoyancy and mobility in fluid. We defined an accurate injection by the presence of a positive air arthrosonogram (see Fig. 1), i.e. the presence of any mobile air visible within the joint cavity at the supra-patellar pouch. The technique was adapted from Qvistgaard et al [20] and applied as follows: at the time of injection, the US probe was applied by GH to the midline suprapatellar pouch in the transverse plane and a video clip recorded. Immediately after the injection, a systematic US of all suprapatellar pouch areas was performed, starting at the site of injection in order to identify a positive air-arthrosonogram. Representative video clips were recorded, as was the sonographer’s immediate impression of whether air-arthrosonogram was present. A positive air-arthrosonogram at any of these sites was used for further analysis. Following intervention, participants were advised to rest the joint as much as possible for 24 h, in line with normal departmental practice.

### Assessment of response

Participants completed the WOMAC pain subscale in clinic at three weeks post injection and again at nine weeks by postal questionnaire. We classified participants as responders at each time point, if their pain scores reduced by 40% or more from baseline, which has been estimated both a clinically important and perceptible change following intervention in knee OA [21].

### Analysis

Descriptive statistics were used to summarise the subject characteristics. We determined at both 3 and 9 weeks whether subjects were responders or non-responders. We looked for differences in subject characteristics by response status at 3 and 9 weeks using t-tests for continuous data and either Fisher’s exact test/Chi-Squared tests for categorical data. Where subjects had omitted individual WOMAC questionnaire items, these were replaced with mean values for the relevant subscale, as far as was permitted according to the manual. Factors considered as potential confounders for adjustment in a multivariable analysis included included gender, baseline pain, radiographic grade, previous injection, body mass index. Inter-observer reliability of the air-arthrosonogram technique was calculated by presenting a sample of 26 stored video clips from the study to three observers (GH, AS, RK; the latter being a consultant radiologist and rheumatologist, respectively, with over ten years experience in musculoskeletal ultrasound each. An equal number of clips were selected from each set of originally classified as positive or negative air arthrograms, respectively, at random. The studies were viewed in random order, independently, by the observers, who then scoring each as showing either positive or negative air-arthrosonogram. Inter-observer agreement was calculated for each pair of observers as a kappa values and the mean value calculated for the three pairs of observers. All analyses were conducted using SPSS version 20.

## Results

### Subjects

A total of 105 patients were recruited to the study (Table 1). Their mean age was 63.1 years and 62 (59%) were female. Mean Body Mass Index (BMI) was 30.8 kg/m^2^. Baseline pain level was 266 (SD 95.5)/500 mm. 104 subjects contributed outcome data at three weeks and 94 (89.5%) at nine weeks. One participant submitted incomplete WOMAC pain scores at 3 weeks. Of the 11 participants who did not contribute to outcome data at nine weeks, one was withdrawn due to an intercurrent episode of crystal arthritis, one questionnaire was lost in the post and 9 failed to return questionnaires. Those who failed to provide data at nine weeks were younger, more likely to be male and had higher CRP values than those providing full data, but there was no difference in accuracy of injections and response rate at 3 weeks between these two groups (see Table 2).

**Table 1:** Baseline characteristics and responder status of study population

Characteristics	n = 105
Age [years] [mean (SD)]	63.1 (11.3)
Female [n (%)]	62 (59%)
BMI [mean (SD)]	30.8 (5.5)
Previous injection [n (%)]	60 (57%)
Baseline WOMAC Pain [mean(SD)]	266 (95.5)
US effusion depth [mm] [Mean (SD)]	3.74 (2.27)
PD grade [median (IQR)]	0 (0,1)
Osteophyte grade [median (IQR)]	2 (1, 3)
JSN grade [median (IQR)]	2 (1,2)
hs CRP [mg/L] [median (IQR)]	4.4 (1.7,7.1)
Positive US arthrogram [n (%)]	74 (70.5%)
Responders at 3 weeks [n (%)]	63 (60.6%)
Responder at 9 weeks [n (%)]	43 (45.7%)

*Abbreviations*: *BMI* body mass index, *PD* power Doppler, *JSN* joint space narrowing, *hsCRP* high-sensitivity CRP, *US* ultrasound

**Table 2:** Characteristics of subjects completing the study compared with those who failed to complete the study

	3 weeks (n = 104)		
Characteristics	Completer (n = 94)	Non completion (n = 11)	*P*
Age [years] [mean (SD)]	64.3 (11.4)	57.1 (8.3)	0.043
Female [n (%)]	59 (62.8%)	3 (27.3%)	0.047
BMI [mean(SD)]	30.7 (5.3)	31.7 (7.5)	0.549
Previous injection [n (%)]	56 (59.6%)	4 (36.4%)	0.199
Baseline Pain [mean (SD)]	264.5 (97.1)	275.4 (84.3)	0.724
US effusion depth [mm] [mean (SD)]	3.7 (2.3)	3.8 (1.7)	0.924
PD grade [median (IQR)]	0 (0,1)	0 (0,1)	0.702
Osteophyte grade [median (IQR)]	2 (1,3)	2 (1,3)	0.977
JSN grade [median (IQR)]	2 (1,2)	2 (1,2)	0.538
hsCRP [mg/L] [median (IQR)]	5.58 (1.5,6.5)	7.80 (3.8, 10.1)	0.021
Positive US arthrogram [n (%)]	66 (70.2%)	8 (72.7%)	1.00
Responder at 3 weeks [n (%)]	56 (60.2)	7 (63.6)	1.00

### Accuracy of injection

Mean inter-observer agreement for air-arthrosonogram status was good, with mean kappa value between three raters of 0.79. 98 (69.5%) injections were considered to be accurate as determined by the presence of air on the arthrosonogram at the time of injection.

### Predictors of response

The overall positive response rate was 61% at three weeks and 46% at nine weeks. A positive arthrosonogram did not predict responder status at 3 or 9 weeks; in fact at both time points, response rates were higher in those with negative than those with positive arthrosonogram (e.g. 75% vs 64%, *p* = 0.365 at nine weeks) (Table 3), although these results were not statistically significant. There were no statistically significant differences in baseline clinical, radiographic, sonographic and serological characteristics between responders and non-responders with two exceptions: Female gender associated with response at three weeks (*p* = 0.045) and previous injection with non-response at nine weeks (*p* = 0.021) (Table 3).

**Table 3:** Baseline characteristics of responders vs non-responders (n = 105)

	3 weeks (n = 104)			9 weeks (n = 94)		
Characteristics	Responder (n = 63)	Non-responder (n = 41)	*P*	Responders (n = 43)	Non-Responder (n = 51)	*P*
Age [years] [mean (SD)]	64.4(10.6)	61.8(11.9)	0.258	64.1(11)	64.5(11)	0.890
Female [n (%)]	42 (67%)	19 (46%)	0.045	31 (72%)	28 (55%)	0.093
BMI [mean(SD)]	31.3(5.4)	30.0(5.8)	0.265	30.9(4.8)	30.4(5.8)	0.658
Previous injection [n (%)]	33 (52%)	26 (63%)	0.314	20 (47%)	36 (71%)	0.021
Baseline Pain [mean (SD)]	256(87)	279(107)	0.240	243(91)	282(99)	0.057
US effusion depth [mm] [mean (SD)]	3.57(2.1)	4.07(2.6)	0.280	3.29(1.9)	4.14(2.59)	0.080
PD grade [median (IQR)]	0(0,1)	0(0,1)	0.790	0(0,1)	0(0,1)	0.376
Osteophyte grade [median (IQR)]	2(1,3)	2(1,3)	0.870	2(1,2)	2(1,3)	0.540
JSN grade [median (IQR)]	2(1,2)	2(1,2)	0.796	2(1,2)	2(1,2)	0.461
hsCRP [mg/L] [median (IQR)]	3.5(1.5,6.8)	4.6(2.2,8.6)	0.147	3.0(1.5,5.7)	4.5(1.7,8.6)	0.857
Positive US arthrogram [n (%)]	42 (67%)	30 (73%)	0.389	27 (64%)	38 (75%)	0.365

See legend to Table 1 for abbreviations

A post-hoc analysis of absolute change in pain scores from baseline in groups with positive vs negative arthrosonogram showed no difference: -114.0 vs -123.4. mm, *p* 0.724 at three weeks; and -58.8 vs -96.5 mm, *p* 0.185 at nine weeks (data not shown in table).

## Discussion

In this prospective observational study we have shown that in knee osteoarthritis accurate intra-articular placement of a corticosteroid injection, as determined by positive air-arthrosonogram, did not improve the rate of clinically significant response or mean pain reduction at three or nine weeks post injection, compared to a group in whom a negative arthrosonogram suggested that placement of injection was predominantly extra-articular. A recent systematic review [13] found that on average only 77% of knee injections enter accurately the intra-articular cavity when using clinical landmarks as guidance for injection. It is therefore plausible to assume that accuracy of injection may potentially explain the variability of response to IACI in knee OA. While there is little doubt that guidance by US improves accuracy of intra-articular placement of injection, there remains uncertainty, however, as to how whether this affects outcome [13]. In a small randomized placebo-controlled study of 38 patients with knee OA, Sambrook et al found no difference in outcome between intentional intra-articular vs peri-patellar injection of methylprednislone between one and 12 weeks post injection. This remains the only study that we are aware of that used clinical guidance of injection to address the role of intra- vs extra-articular corticosteroid injection in knee OA on outcome. The fact that no difference was observed in that study may have been due to the fact that correct intra-articular placement was not verified by further imaging (i.e. some intended intra-articular injections may still have ended up extra-articularly) and/or lack of power (i.e. insufficient study sample) to show a difference. Sibbitt et al [22] examined the effect of ultrasound (US)-guidance vs clinically guided IACI in a randomised study of 94 subjects with knee OA. No information on accuracy of the injection with either method is provided, but subjects undergoing US-guided injection were approximately twice as likely to have a positive response at 2 weeks (defined as a pain VAS of < 2 cm). A second smaller study of essentially similar design by the same group showed similar findings. Both studies have not tried to control for any treatment effect likely to be conferred by the use of ultrasound itself, in other words: having an injection under ultrasound than without may have significant beneficial effect per se without any relationship to improved accuracy. That this is likely to be relevant is suggested by work by Cunnington et al [23] who used sham-US to compare US-guided injection vs clinically-guided injections in a variety of joints in a large study of 184 subjects with inflammatory arthritis: whilst, as expected US-guided injections were more accurate than clinically guided with sham-US, there was no difference in outcome (both pain and function) between the two groups. Furthermore, and more relevant to this study, function (but not pain) at six (but not two) weeks was the only parameter that showed positive correlation with accuracy of injection. A trial studying US-guided vs ‘sham-US’ clinically-guided injection has been terminated and is to date unreported (ClinicalTrials.gov Identifier: NCT01032720; accessed 15 June 2015).

We chose outcome assessment at three and nine weeks, as these time points reflect the period during which benefit of treatment has been suggested in previous systematic reviews of placebo-controlled trials [5, 6].

Our study showed further interesting observations: participants who had previous experience of injection were less likely to report response to treatment than those undergoing their first injection at nine weeks (*p* 0.021), but not three weeks. This seems to concur with the anecdotal clinical observation that the effect of subsequent IACI in knee OA is less than of first injections. In line with most [11, 24–26], but not all studies [9, 10], we found no association between radiographic severity and rate of response, despite scoring separately for individual features of knee osteoarthritis. In accordance with other investigators [27, 28], we found no relationship between response and sonographic effusion or synovial power Doppler, despite using a sonographic assessment that included measures from the medial, mid and lateral aspect of the supra-patellar pouch rather than single view data.

The main strength of our study is that it was based on routine practice of IACI, using clinical guidance and subjects referred for IACI by their clinician as part of routine clinical management. As such we believe the results are applicable to a general population. However, we have seen a better response rates than placebo-controlled studies [5] probably due to contextual, non-specific treatment effects. Conversely, in trying to reflect a ‘real-world’ treatment environment, we did not limit the use of other interventions including analgesics, walking sticks and physiotherapy, which could have affected patient outcomes.

This study has a number of limitations: As an observational study, it remains possible that an adequately powered, placebo-controlled trial might disclose an effect of accuracy of injection on outcome. We did not perform a formal power calculation based on arthrogram status for this observational trial and one could argue that our sample size is too small to show an effect of accuracy or other structural factors on outcome However, the reported rate of response was numerically higher in those with a negative air-arthrosonogram at both three and nine weeks, suggesting that a larger sample would be unlikely to show superiority of response rate in those with a positive vs those with a negative arthrogram. We did not use a second imaging method (e.g. MRI or limited CT) to validate our method to verify accurate intra-articular injection as previously described by Qvistgaard et al [20]. We therefore acknowledge that there are limitations in the air-arthrosonogram technique as an indicator of intra- vs extra-articular placement of injection, even though our interobserver reliability was good. Our accuracy data on the basis of the air-arthrosonogram method of 70% are also broadly in line with those reported in a recent systematic review [13]. We therefore do not believe that the technique resulted in significant systemic misclassification.

Beyond the question of relevance of precise intra- vs extra-knee joint cavity placement, the question remains as to whether an injection close to the synovium (e.g. the fat-pad of the knee) can be shown to be of equivalent effectiveness on outcome. Our study suggests this possibility, but due to its lack of precise visualisation of injectate localisation in those with a negative arthrosonogram, we are unable to draw any firm conclusions on this possibility. Our findings suggest that accurate intra-articular injection is neither a guarantor nor a pre-requisite of response at 3 and 9 weeks and provides support the current practice of IACI in knee OA using clinical landmarks and palpation. Whilst US may well have a treatment effect of its own and recognised to be useful to assist for aspiration or technically difficult injections, there is to date no convincing indication that the improved accuracy it confers to the injection matters to outcome of pain relief in this setting.

## Conclusion

Our ‘real-life’, observational study suggests that IACI based on routine, clinically guided practice leads to relief of knee pain in osteoarthritis in the short- and medium-term in patients with uncertain placement of injection, as well as those in whom injections could be demonstrated to be in the intra-articular space. This casts doubt over the relevance of accuracy of intra-articular injection placement to clinical outcome in this setting.

## Abbreviations

ACR: American college of rheumatology
BMI: Body mass index
IACI: Intra-articular corticosteroid injections
IQR: inter-quartile range
JSN: Joint space narrowing
KOA: knee osteoarthritis
PD: Power doppler
RA: Rheumatoid arthritis
SD: Standard deviation
US: ultrasound
VAS: Visual analogue score
WOMAC: Western Ontario and McMaster Universities Osteoarthritis Index
